# Wheat-Based Protein Slows Disease Progression in *Pkd1* Knockout Mice

**DOI:** 10.1093/function/zqaf026

**Published:** 2025-06-10

**Authors:** Randee Sedaka, Jifeng Huang, Shinobu Yamaguchi, Emily Hallit, Aida Moran-Reyna, Jung-Shan Hsu, Caleb Lovelady, Ayaka Fujihashi, Mohammad Sako, Malgorzata Kasztan, Gloria Benavides, Landon Wilson, Victor Darley-Usmar, Stephen Barnes, Takamitsu Saigusa

**Affiliations:** Section of Cardio-Renal Physiology and Medicine, Division of Nephrology, Department of Medicine, University of Alabama at Birmingham, Birmingham, AL 35294, USA; Section of Cardio-Renal Physiology and Medicine, Division of Nephrology, Department of Medicine, University of Alabama at Birmingham, Birmingham, AL 35294, USA; Section of Cardio-Renal Physiology and Medicine, Division of Nephrology, Department of Medicine, University of Alabama at Birmingham, Birmingham, AL 35294, USA; Section of Cardio-Renal Physiology and Medicine, Division of Nephrology, Department of Medicine, University of Alabama at Birmingham, Birmingham, AL 35294, USA; Section of Cardio-Renal Physiology and Medicine, Division of Nephrology, Department of Medicine, University of Alabama at Birmingham, Birmingham, AL 35294, USA; Section of Cardio-Renal Physiology and Medicine, Division of Nephrology, Department of Medicine, University of Alabama at Birmingham, Birmingham, AL 35294, USA; Section of Cardio-Renal Physiology and Medicine, Division of Nephrology, Department of Medicine, University of Alabama at Birmingham, Birmingham, AL 35294, USA; Section of Cardio-Renal Physiology and Medicine, Division of Nephrology, Department of Medicine, University of Alabama at Birmingham, Birmingham, AL 35294, USA; Division of Pediatric Hematology/Oncology, Department of Pediatrics, University of Alabama at Birmingham, Birmingham, AL 35294, USA; Division of Pediatric Hematology/Oncology, Department of Pediatrics, University of Alabama at Birmingham, Birmingham, AL 35294, USA; Department of Pathology, University of Alabama at Birmingham, Birmingham, AL 35294, USA; Targeted Metabolomics and Proteomics Laboratory, University of Alabama at Birmingham, Birmingham, AL 35294, USA; Department of Pathology, University of Alabama at Birmingham, Birmingham, AL 35294, USA; Targeted Metabolomics and Proteomics Laboratory, University of Alabama at Birmingham, Birmingham, AL 35294, USA; Section of Cardio-Renal Physiology and Medicine, Division of Nephrology, Department of Medicine, University of Alabama at Birmingham, Birmingham, AL 35294, USA

**Keywords:** ADPKD, plant protein, lysine, animal protein, metabolomics, glutamine

## Abstract

Dietary load and composition are known contributors that accelerate cyst growth in polycystic kidney disease (PKD). High protein intake, which increases amino acid burden in the kidneys, is one such factor. Despite identical protein load, a plant-based wheat-gluten (WG) diet was recently reported to blunt the inflammatory response of animal-based casein diet in a hypertensive model. Considering the importance of pro-inflammatory signals on cystogenesis in PKD, we therefore sought to determine whether a WG compared to casein diet would decelerate cyst progression. Tamoxifen-inducible, global *Pkd1* knockout mice were fed either a low casein (6%), high casein (60%), or high wheat-gluten (60%) protein diet for 6 wk. In a separate cohort, mice were gavaged daily with vehicle, lysine, or glutamine for 4 wk while maintained on a normal protein (18%) diet. Tissues were used for histology, flow cytometry, mitochondrial function, metabolomics, and various biochemical assays. WG-fed mice had better kidney function and reduced kidney macrophage percentages, proinflammatory cytokine expression, and cyst growth compared to casein-fed mice. Protein source did not alter kidney mitochondria function. Supplementation with lysine, the highest amino acid in casein versus WG diet, increased kidney cyst growth, acid production, and metabolic disarray. This did not occur with glutamine supplementation, the highest amino acid in WG versus casein diet, despite increased glomerular filtration rate with both amino acids. Neither supplementation mounted an inflammatory response. A plant-based, low-lysine diet slows disease burden in a murine model of PKD. This easily modifiable diet may be a beneficial intervention for PKD patients.

## Graphical Abstract

**Graphical Abstract:**
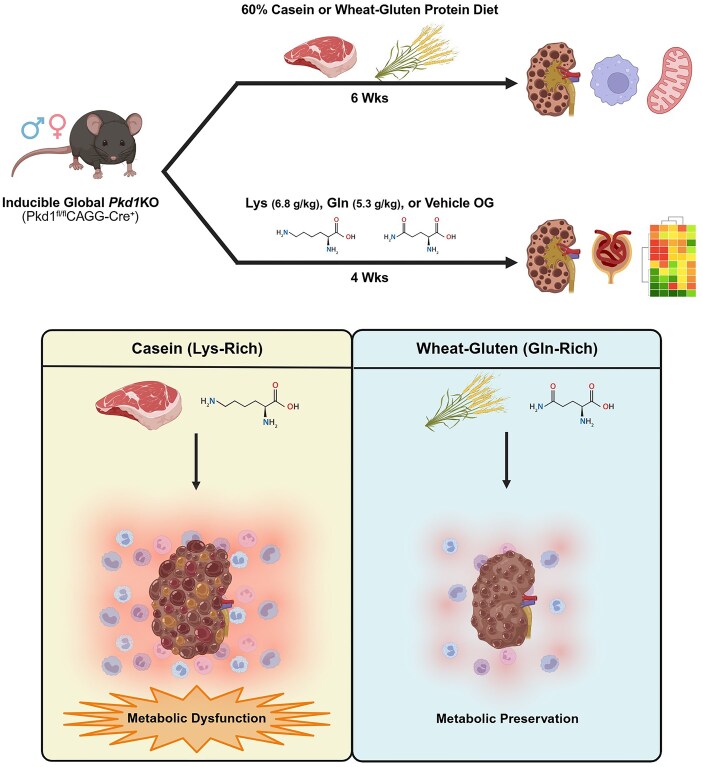

## Introduction

Autosomal dominant polycystic kidney disease (ADPKD) is a genetic disorder, progression of which is modifiable by environmental factors.^1-5^ Compelling evidence suggests that compensatory renal hypertrophy, triggered by unilateral nephrectomy^3,6,7^ or high protein intake,^8,9^ exacerbates cyst progression in rodents. This hypertrophic response involves kidney hyperfiltration^10^ and thus increased tubular delivery of amino acids and humoral factors.^11^ We recently reported that a chronic, animal-based protein load accelerates cystogenesis in *Pkd1* knockout (*Pkd1*KO) mice via early metabolic alterations, including increased glutamine transport, prior to a rise in kidney macrophage (MФ) populations.^12^ Metabolic dysfunction, such as glutamine-reliance to fuel the citric acid (TCA) cycle, is prevalent with loss of *Pkd1*,^13-16^ whereby a number of individual amino acids have been reported to influence cystogenesis.^17-20^

Plant-based soy protein has been extensively studied as a replacement to animal-based casein protein in PKD rodents. Unfortunately, results of these studies were variable, slowing kidney cyst growth in non-orthologous,^21-23^ but not orthologous, PKD models.^24,25^ Furthermore, the phytoestrogens in soy stimulated liver cyst growth due to estrogen receptor localization in liver cyst epithelia.^26^ Wheat-gluten (WG), another plant-based protein which does not contain phytoestrogen, was recently reported to mitigate high-salt induced hypertension, kidney immune cell activation, and tubular injury in rats compared to casein.^27^ Therefore, we aimed to investigate whether a WG protein-load would slow PKD progression compared to a casein load in our *Pkd1*KO mice.

Here, we report that high dietary WG in lieu of casein improves kidney function and stunts growth while reducing kidney MФ abundance and inflammatory cytokines in PKD. *Pkd1*KO mice fed a normal protein diet with oral supplementation of lysine (rich in casein) compared to glutamine (rich in WG) had more cystic kidneys, acidosis, and metabolic stress without inflammatory changes. Specifically, lysine treatment lead to a reduction in various TCA cycle-related metabolites, which was not observed with long-term glutamine supplementation. Thus, plant-based protein may have cyst-slowing effects, while lysine contributes to animal-based protein kidney damage in PKD.

## Materials and Methods

### Animals

Global, conditional *Pkd1* knockout (*Pkd1*KO) mice were generated and maintained as previously reported.^12^ Six-week-old male and female *Pkd1*KO mice were intraperitoneally injected with Tamoxifen (T5648, MilliporeSigma, Burlington, MA; 9 mg/40 g in corn oil every other day, 3 doses) to induce PKD. Efficient knockout was confirmed by kidney *Pkd1* expression. A 12:12 h light-dark cycle was used under constant temperature and humidity with food and water provided *ad libitum*. Mice were randomly assigned to dietary interventions, utilizing 125 total mice. Kidney and spleen tissue were harvested under constant isoflurane inhalation followed by thoracotomy. Each data point signifies one mouse. Experimental protocols were approved by the Institutional Animal Care and Use Committee at the University of Alabama at Birmingham, performed in accordance with the National Institutes of Health Guide for the Care and Use of Laboratory Animals, and reported in agreement with Animal Research: Reporting of *In Vivo* Experiments (ARRIVE) guidelines (https://arriveguidelines.org).

### Dietary Intervention

Eight-week-old mice were placed on a low casein (LC; 6%; TD.90016), high casein (HC; 60%; TD.6220), or high wheat-gluten (WG; 60%; TD.190147) protein diet for 6 wk *ad libitum*. All diets were formulated by Envigo (Indianapolis, IN, USA) to be isocaloric (3.7 kcal/g). Nutritional and amino acid composition are shown in Supplementary Tables S1 and S2, respectively. In these studies, low protein acts as a control to exemplify the impact of high protein. In a separate cohort, mice received an oral gavage (OG) of glutamine (5.25 g/kg/day; G8540, MilliporeSigma), lysine (6.8 g/kg/day; L8662, MilliporeSigma), or vehicle (1X phosphate-buffered saline; PBS) for 4 wk while maintained on normal protein (18%) chow. To limit potential cofounding factors, supplementation groups were cohoused and OG order alternated daily.

### Cyst Quantification

Kidney tissue was immersed in 10% formalin (S25689, Fisher Scientific, Hampton, NH) prior to paraffin-embedding. Sections (5-μm) were cut and stained with hematoxylin-eosin (H&E). Whole kidney images were captured at 4× magnification using a Keyence BZ-X710 (Itasca, IL, USA) microscope and cysts were blindly quantified using Image J as previously described.^12^

### Flow Cytometry

After cardiac perfusion with 1X PBS, kidneys were minced, digested in collagenase type I containing buffer, and passed through 70-µm mesh to yield a single-cell suspension. Red blood cells were lysed, followed by cell blocking, primary antibody staining (Supplementary Table S3), fixing in 2% paraformaldehyde, and finally resuspended in 1X PBS. Spleens were used as single color staining controls. The gating strategy to identify infiltrating (CD11b^hi^, F4/80^lo^) versus tissue-resident (CD11b^lo^, F4/80^hi^) macrophages was previously described.^12^ Cells were analyzed on a BD LSRII flow cytometer and data analysis was performed using FlowJo v10 software.

### Quantitative Real-Time PCR

RNA was isolated from whole kidney tissue using TRIzol Reagent (15-596-018, Fisher Scientific) and 1.0 μg was reverse transcribed using Bio-Rad’s iScript cDNA Synthesis Kit (1 708 891, Hercules, CA, USA) or Applied Biosystems’ High-Capacity cDNA Reverse Transcription Kit (4 368 814, Fisher Scientific). Relative gene expression was measured on a CFX96 Touch Real-Time PCR Detection System (Bio-Rad) using SsoAdvanced Universal SYBR Green Supermix (1725271, Bio-Rad) and primers synthesized by Integrated DNA Technologies (Supplemental Table S4; Coralville, IA, USA) or TaqMan Gene Expression Master Mix (4369514, Fisher Scientific) and Taqman probes for *Ccl2* (Mm00441242), *Csf1* (Mm00432686), *Il-6* (Mm00446190), *Tgfb1* (Mm01188201), *Cxcl2* (Mm00436450), *Kim1* (Mm00506686), and *Gapdh* (4352339E, Fisher Scientific). Data were normalized to *Gapdh* and relative to low casein or vehicle groups.

### Mitochondrial Respiratory Complex Activity

Kidney tissue from high casein or WG diet fed mice was flash frozen and pulverized before mechanical glass-glass homogenization in MAS buffer (70 m m sucrose, 220 m m mannitol, 5 m m KH2PO4, 5 m m MgCl2, 1 m m EGTA, 2 m m HEPES pH 7.4). Samples were centrifuged at 1000 *g* for 10 min at 4°C and supernatants were kept to measure protein concentration and complex activity. Kidney homogenates (4 µg protein/20 µL MAS buffer) were loaded into a Seahorse XF96 microplate and centrifuged at 2000 *g* for 20 min at 4°C. MAS containing cytochrome c (10 µm) and alamethicin (10 µg/mL) was added to each well. To determine specific complex activity, the following substrates and inhibitors were used^28,29^: Complex I- NADH (1 m m), then rotenone (2 µm); Complex II- succinate (5 m m) + rotenone (2 µm), then antimycin A (10 µm); Complex III- duroquinol (0.5 m m), then antimycin A (10 µm); Complex IV- Tetramethylphenylenediamine (TMPD; 0.5 m m) + ascorbic acid (1 m m), then azide (20 m m). Data were normalized to tissue protein.

### Western Blot

Kidney tissue samples were homogenized and probed as previously described.^12^ Membranes were blocked with EveryBlot Blocking Buffer (12010020, Bio-Rad Laboratories, Inc., Hercules, CA, USA) for 5 min followed by a 4°C overnight incubation with Cell Signaling (Danvers, MA, USA) phospho-S6 ribosomal protein (Ser240/244; 5364, 1:500) or phospho-AMPKα (Thr172; 2535, 1:100) primary antibody and goat anti-rabbit IgG, DyLight 680 (35569, Fisher Scientific) secondary for 1 h at room temperature. Equal protein loading was verified by β-actin staining (3700, 1:10 000 Cell Signaling; followed by goat anti-mouse IgG, DyLight 800 [SA5-10176], Fisher Scientific) and shown in each representative figure. Only male samples were assessed as female mice were not responsive to changes in protein source.

### Glomerular Filtration Rate (GFR)

GFR was measured at baseline and after chronic OG administration using a transcutaneous measurement of fluorescein isothiocyanate-labeled sinistrin (Fresenius Kabi Austria, Linz, Austria) as previously described.^30^ Briefly, mice were intravenously injected with sinistrin (0.15 mg/g body weight) and fluorescent signal was recorded (3 h) via a non-invasive clearance device (MediBeacon, Mannheim, Germany). GFR (μL/min/100 g body weight) was assessed by a blinded technician. Delta GFR was calculated by subtracting baseline from OG values.

### Blood and Urine Analyses

Blood removed by cardiac puncture with 0.5 M EDTA (15-575-020, Fisher Scientific) coated needles was centrifuged at 2000 *g* for 10 min to collect plasma. Creatinine was measured using isotope dilution liquid chromatography-tandem mass spectrometry while glucagon was measured by ELISA (81 518; Crystal Chem, Elk Grove Village, IL) following the manufacturer’s protocol. In a separate cohort, blood was removed using heparin-coated needles and immediately applied to an Abbott Laboratories Blood Gas and Electrolytes i-STAT EC8 + Cartridge (03P7925, Chicago, IL) to measure blood urea nitrogen (BUN), pH, bicarbonate (HCO3), and glucose. Remaining blood was centrifuged as above to collect plasma.

Ammonia was measured in spot urine using Cell Biolabs, Inc. colorimetric assay (MET-5086, San Diego, CA, USA) according to the manufacturer’s protocol and normalized to protein content measured via Bradford assay (5000205; Bio-Rad).

### Liquid Chromatography Tandem Mass Spectrometry

Kidneys cortex tissue (100 mg) was bead-homogenized, then extracted using the Bligh-Dyer chloroform: methanol procedure.^31^ Briefly, the upper aqueous layer was transferred to a glass tube with cold methanol (80%) to further remove proteins. After centrifugation, supernatants were dried, re-suspended in 0.1% formic acid, and analyzed by Liquid Chromatography Tandem Mass Spectrometry using a SCIEX 5600 TripleTOF mass spectrometer for untargeted metabolomics analyses (positive and negative ionization modes). Metabolites were resolved using a Phenomenex 2.1 i.d. ×100 mm, LunaOmega 1.6 μm C_18_ reverse-phase column with a linear gradient of 2-50% acetonitrile in 0.1% formic acid over 5 min at 500 μL/min. Data were analyzed using MetaboAnalyst 6.0. See supplement for full protocol.

### Statistical Analyses

Data are presented as means ± SEM. Differences between groups were analyzed using GraphPad Prism 10 (La Jolla, CA, USA) by Ordinary one-way ANOVA with Tukey’s multiple comparisons test, two-tailed unpaired Student’s *t*-test, or two-way ANOVA with Tukey’s multiple comparisons test, as noted. *P* < 0.05 denotes statistical significance. Experiments were conducted and analyzed by independent individuals to limit bias in outcomes.

## Results

### Wheat-gluten Versus Casein Protein Diet Improves Kidney Function and Reduces Inflammation in PKD

**Figure 1.**
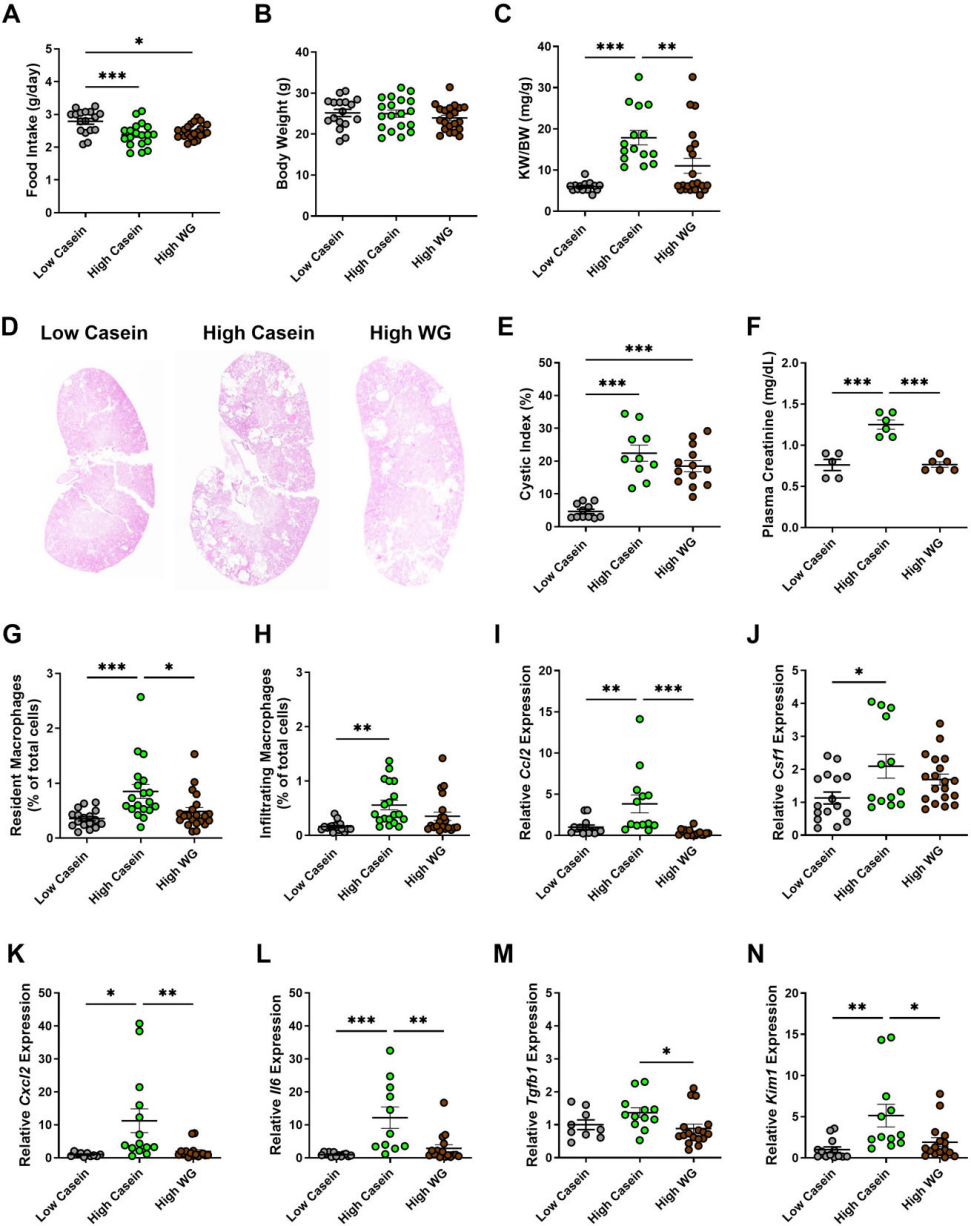
The high-protein-induced inflammatory response in PKD mice is partially mitigated by wheat gluten diet. **(A)** Average daily food intake during 6-wk dietary intervention. *Pkd1*KO mouse ate more low casein protein versus high protein casein or wheat gluten (WG) diet. **(B)** Body weight was not different amongst genotypes. **(C)** Kidney weight to body weight ratio (KW/BW). *Pkd1KO* mice fed a high casein diet had elevated KW/BW ratio compared to those fed low casein or high WG diet. **(D-E)** Representative *Pkd1*KO kidney histology and cystic index. Cystic index was elevated with high protein consumption regardless of source. **(F)** Plasma creatinine elevated with high casein intake versus low casein or WG. **(G-H)** Number of resident and infiltrating macrophages. Both macrophage populations increase with high casein diet, but resident macrophages decrease with high WG diet. **(I-N)** Kidney gene expression of *Ccl2, Csf1, Cxcl2, Il6, Tgfb1*, and *Kim1*. High casein stimulated *Ccl2, Csf1, Cxcl2, Il6*, and *Kim1* expression compared to low casein, while WG diet reduced *Ccl2, Cxcl2, Il6, Tgfb1*, and *Kim1* expression compared to high casein diet in *Pkd1*KO mice. (*n* = 6-21/group). Results of a One-way ANOVA with Tukey's multiple comparisons test reported for all. *P* *< 0.05, **< 0.01, ***<0.001.

To determine the effect of a plant-based diet on cyst growth and immune response, 8-wk-old *Pkd1*KO mice were fed either a high WG, HC, or LC diet for 6 wk. Average daily food intake was higher in LC compared to WG and HC fed mice, though intake was similar between high protein fed mice (Figure 1A). After dietary intervention, there were no differences in body weight between LC, HC, or WG fed mice (Figure 1B), but kidney weight (average of 2 kidneys) to body weight (KW/BW) ratio was elevated in HC-fed mice and attenuated by WG diet (Figure 1C). Cystic index was elevated in HC versus LC fed mice (Figure 1D-E). Although WG did not mitigate cyst development overall, it did improve outcomes in male, but not female, mice (Supplementary Fig. S1A, S1D). Plasma creatinine concentration was higher in HC compared to WG and LC fed mice (Figure 1F).

HC-fed *Pkd1*KO mice had a higher percentage of kidney resident (Figure 1G) and infiltrating (Figure 1H) MФs compared to LC-fed counterparts. WG diet only blunted the resident MФ response. Similar to cystic index, however, switching to a WG diet resulted in fewer resident and infiltrating MФs in males (Supplementary Fig. S1B-C), but not females (Supplementary Fig. S1E-F). To further evaluate the potential inflammatory benefits of WG, we quantified gene expression of various kidney cytokines/chemokines. Consuming a HC diet increased the expression of MФ-recruiting chemokine *Ccl2* (Figure 1I), MФ-expanding cytokine *Csf1* (Figure 1J), chemo-attractant cytokine *Cxcl2* (Figure 1K), pro-inflammatory cytokine *Il6* (Figure 1L), and kidney injury marker *Kim1* (Figure 1N) in *Pkd1*KO mice compared to a LC diet. WG dampened the expression of *Ccl2, Cxcl2, Il6*, pro-fibrotic cytokine *Tgfb1* (Figure 1M), and *Kim1* in contrast to HC.

### Protein Source Does Not Affect Kidney Mitochondrial Function

**Figure 2.**
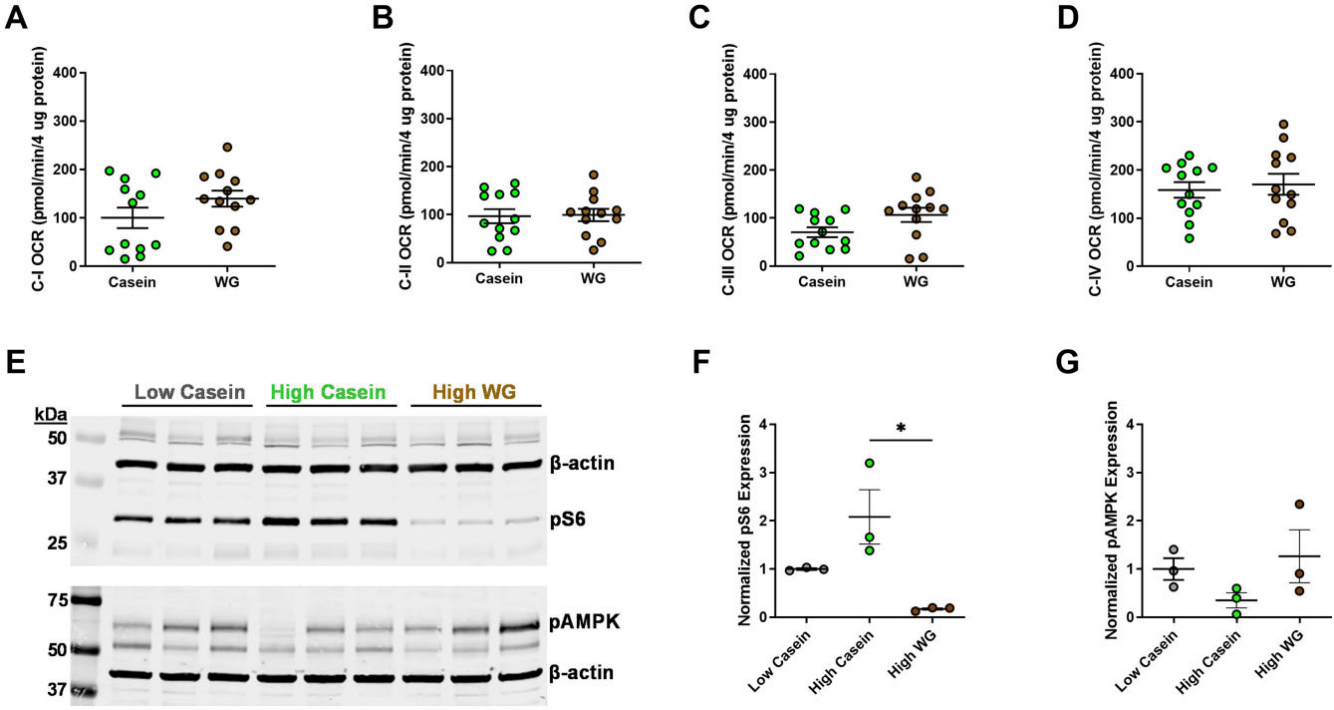
WG diet does not alter mitochondrial metabolism. **(A)** Mitochondrial complex I, **(B)** complex II, **(C)** complex III, and **(D)** complex IV oxygen consumption rate (OCR) between *Pkd1*KO mice fed high casein or WG diet for 6 wk. No differences in OCR were observed between diets (*n* = 12/group). **(E-G)** Representative Western blots and densitometric quantification of kidney phosphorylated S6 (pS6) and phosphorylated adenosine monophosphate-activated protein kinase (pAMPK). High casein feeding increases pS6 and decreases pAMPK compared to low casein and is mitigated by high WG (*n* = 3/group). Results of an unpaired *t* test reported for A-D and Ordinary one-way ANOVA with Tukey's multiple comparisons test reported for E-G, *P < 0.05.

Amino acids are primarily metabolized and utilized in the mitochondria,^32,33^ the function of which is known to be defective in PKD.^34-36^ Therefore, we investigated whether a WG diet could improve mitochondrial function in our *Pkd1*KO mice compared to HC diet. Oxygen consumption rate (OCR) in the kidney was not different between the diets when sexes are combined (Figure 2A**-**D). However, when stratified by sex, males had a higher OCR compared to females regardless of membrane complex (Supplementary Fig. S2). Interestingly, WG diet did improve complex I OCR in female mice. To investigate whether WG modifies other commonly dysregulated pathways in PKD, we measured the abundance of pS6, an activation marker for mTOR during PKD progression, and pAMPK, which suggests mTOR inhibition. WG-fed mice displayed a reduction in pS6 and elevation in pAMPK compared to HC-fed mice, which is akin to the LC-fed mice (Figure 2E-G). These results indicate that dietary wheat-gluten can improve kidney inflammation and injury when substituted for casein (predominantly in males), but does not affect mitochondrial function.

### Dietary Lysine, But Not Glutamine, Increases Acid Production and Accelerates Cyst Growth

Next, we compared the diets to elucidate any composition differences that may contribute to the aforementioned beneficial effects of WG. Major differences amongst the diets regarded sucrose, cellulose, corn oil and protein content. Sucrose, corn oil and cellulose were highest in the LC diet (Supplementary Table S1); however, these components are unlikely to contribute to cyst growth as *Pkd1*KO mice fed a LC diet had the smallest, least cystic kidneys (Figure 1). Focusing on differences in amino acid composition between HC and WG diets, lysine (orange) is most abundant in HC, whereas a combination of glutamine and glutamate (shown as Glu; blue) is most abundant in WG diet (Supplementary Table S2). Thus, we supplemented a normal protein diet with either lysine or glutamine to determine whether either amino acid could alter cyst growth.

**Figure 3.**
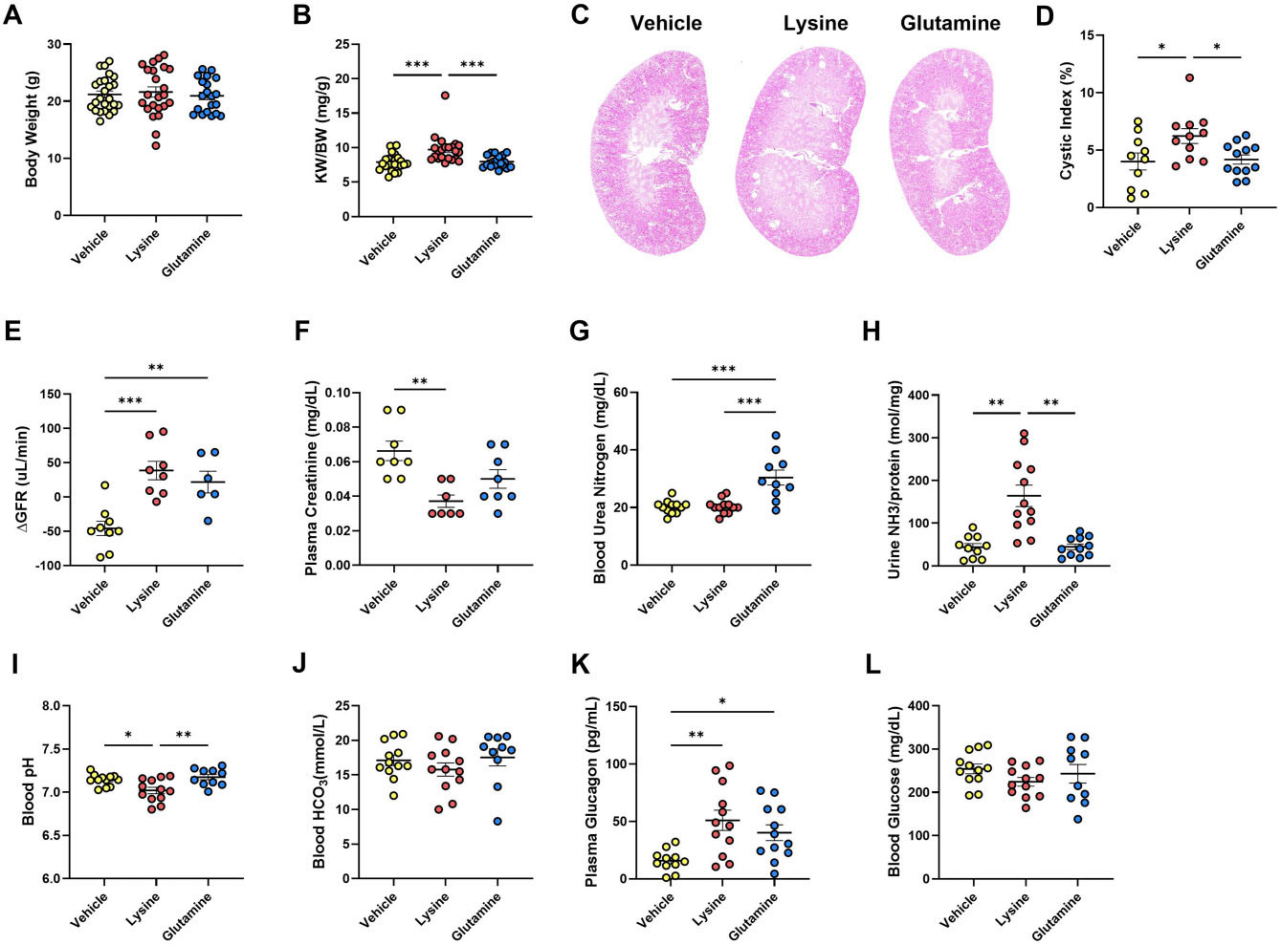
Lysine supplementation increases cystic burden and causes metabolic acidosis in *Pkd1*KO mice. **(A)** Body weight was not different amongst *Pkd1*KO mice given vehicle, glutamine, or lysine by oral gavage (OG) for 4 weeks. **(B)** KW/BW ratio was increased in mice given lysine. **(C-D)** Representative *Pkd1*KO kidney histology and cystic index. Lysine OG increased cystic index compared to vehicle or glutamine dosed mice. **(E)** Change in glomerular filtration rate (GFR) from immediately before to after 4 wk of OG. Glutamine and lysine OG increased GFR. **(F)** Plasma creatinine was lower in lysine versus vehicle OG mice. **(G)** Blood urea nitrogen was elevated in glutamine compared to vehicle or lysine gavaged mice. **(H)** Urinary ammonia was increased, while **(I)** blood pH was decreased with lysine OG. **(J)** No differences were observed in blood bicarbonate between gavage groups. **(K)** Plasma glucagon was elevated by lysine OG, although no differences in **(L)** blood glucose were observed. (*n* = 3-25/group). Results of a One-way ANOVA with Tukey’s multiple comparisons test reported for all. *P* *< 0.05, **< 0.01, ***< 0.001.

Chronic supplementation with lysine had no effect on body weight (Figure 3A), but did increase KW/BW ratio (Figure 3B) and cystic index (Figure 3C-D) in contrast to vehicle or glutamine supplementation. Both amino acids increased GFR compared to vehicle treated mice (Figure 3E), while plasma creatinine was only lower in lysine compared to vehicle supplemented mice (Figure 3F). Blood urea nitrogen (BUN) was higher in glutamine-treated mice compared to vehicle or lysine (Figure 3G) without a dip in plasma creatinine, indicating that the glutamine-based BUN increase is not due to kidney failure. Glutamine metabolism generates ammonia (NH3) in the kidney which gets secreted into the urine. Surprisingly lysine supplementation, but not glutamine or vehicle, increased urinary NH3 excretion (Figure 3H) and lowered blood pH (Figure 3I) without altering total blood bicarbonate (Figure 3J). Plasma glucagon concentration, which increases upon protein or amino acid load, was higher in lysine-supplemented mice compared to vehicle or glutamine (Figure 3K), but there were no differences in blood glucose concentration (Figure 3L).

### Lysine Supplementation Increases Expression of Glucose Metabolism and Glutamine Transport Markers, But Not Inflammation

**Figure 4.**
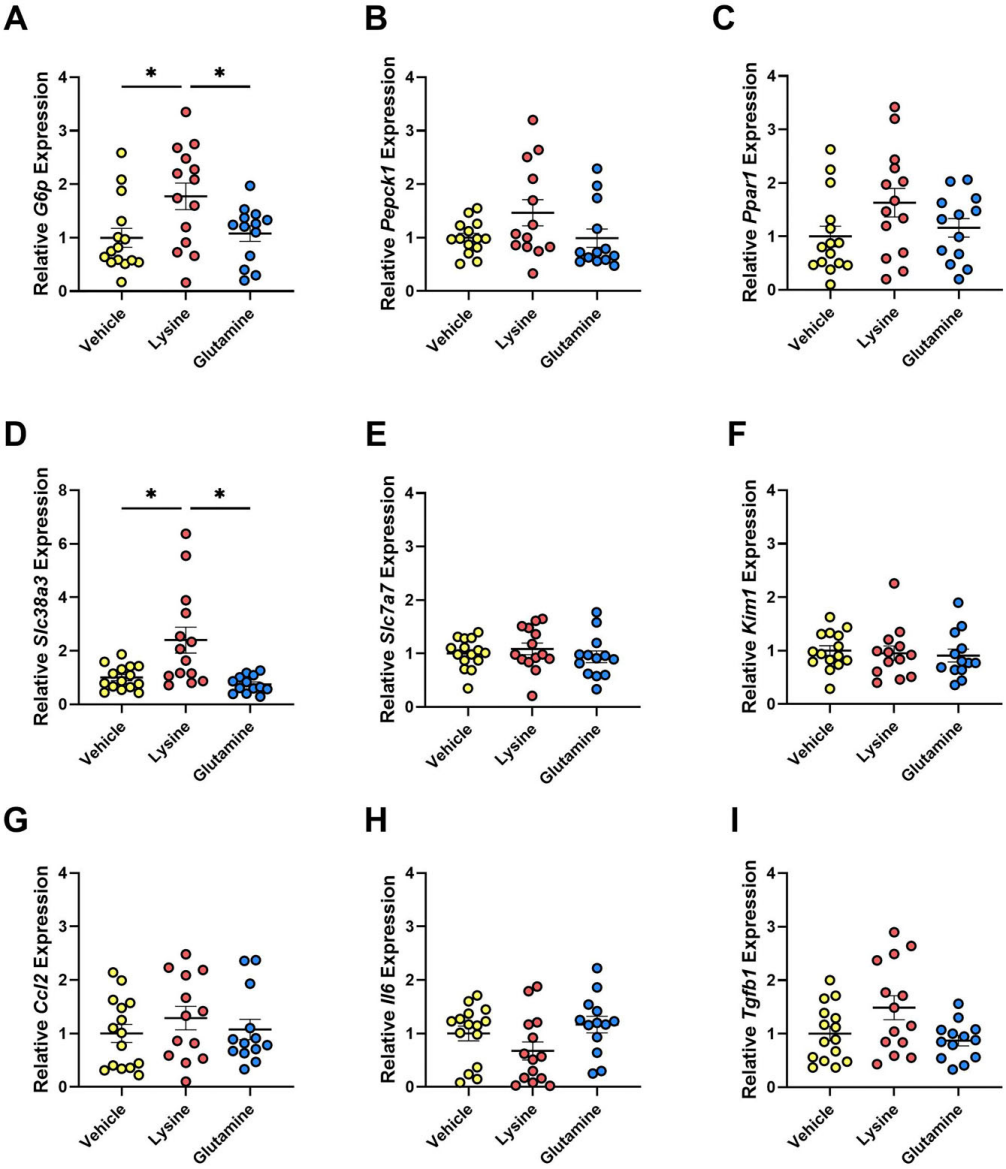
*G6p* and *Slc38a3* expression increases with lysine gavage. **(A)** Kidney expression of *G6p*, **(B)**  *Pepck1*, **(C)**  *Ppar1*, **(D)**  *Slc38a3*, **(E)**  *Slc7a7*, **(F)**  *Kim1*, **(G)**  *Ccl2*, **(H)**  *Il6*, and **(I)**  *Tgfb1* in *Pkd1*KO mice after 4 wk of OG. Lysine treatment increased the expression of kidney *G6p* and *Slc38a3* compared to vehicle or glutamine treatment. (*n* = 13-15/group). Results of a One-way ANOVA with Tukey's multiple comparisons test reported for all. *P* *< 0.05.

Gluconeogenic enzyme glucose-6-phosphatase (*G6p*) (Figure 4A), but not *Pepck1* (Figure 4B), was more highly expressed in *Pkd1*KO mice treated with lysine versus vehicle or glutamine. There were no differences in fatty acid metabolism marker *Ppar1* between groups (Figure 4C). Unexpectedly, *Slc38a3*, a sodium-glutamine transporter, was elevated in lysine-supplemented mice compared to glutamine or vehicle treatment (Figure 4D), which corroborated the increased NH3 excretion (Figure 3H). Expression of amino acid transporters *Slc7a7* (Figure 4E) and *Slc7a9* (not shown), both mediating cellular lysine movement, were similar between treatment groups. Despite the increase in cystic index imparted by lysine supplementation, there were no differences in the expression of *Kim1* (Figure 4F)*, Ccl2* (Figure 4G), *Il6* (Figure 4H), or *Tgfb1* (Figure 4I) between treatment groups in *Pkd1*KO mice. Taken together, this suggests that although dietary glutamine and lysine both increase GFR compared to vehicle, only lysine supplementation accelerates kidney cyst growth, glutamine transporter expression, ammonia excretion, and metabolic acidosis without provoking an inflammatory response.

### Untargeted Metabolomics Pinpoints Lysine-Mediated Metabolic Stress and Sex Differences in *Pkd1*KO Mice

**Figure 5.**
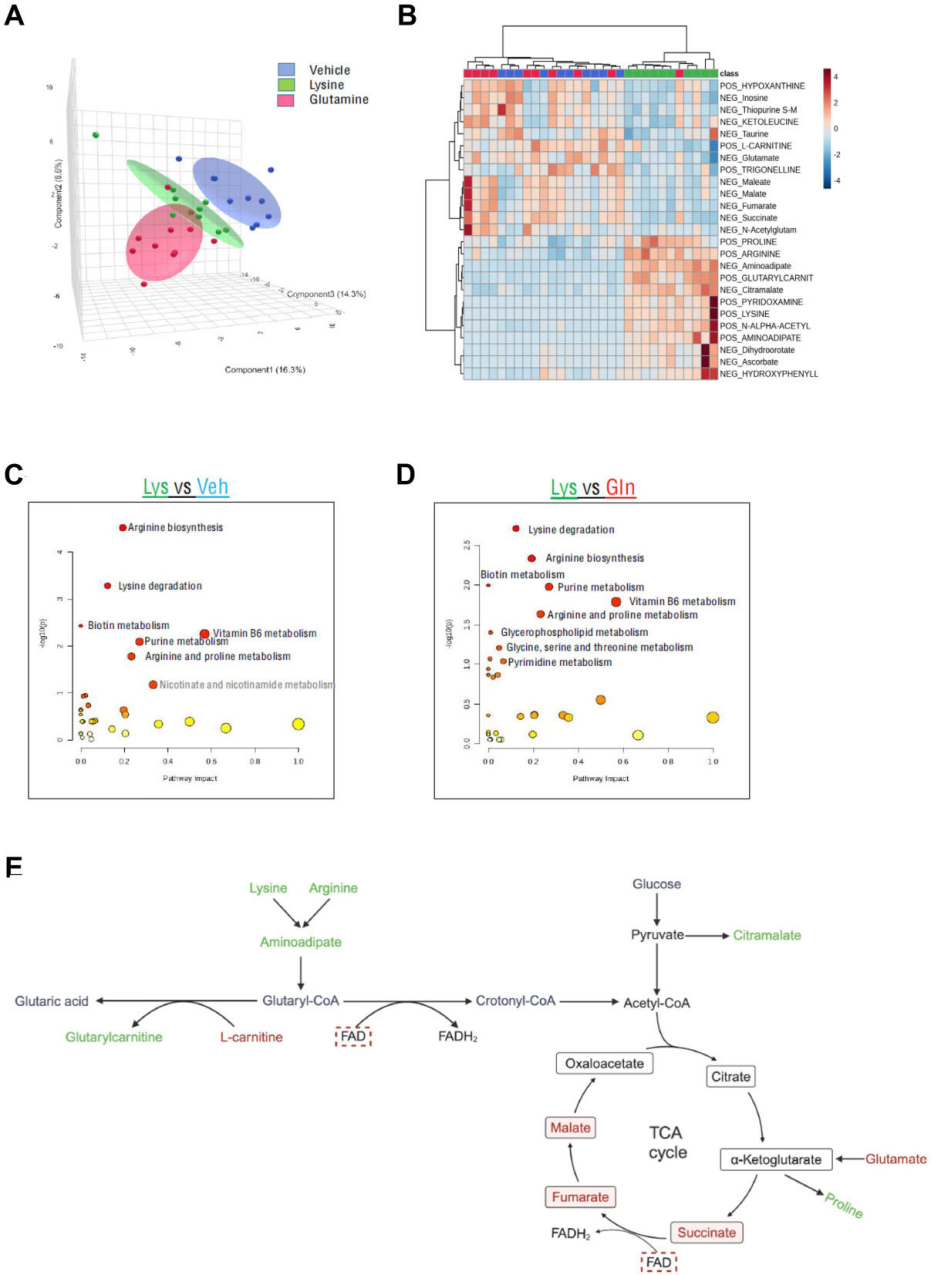
Metabolic stress in lysine-supplemented *Pkd1*KO mice. **(A)** 3D scores plot between the selected primary components with variances shown in differently colored ellipses. **(B)** Metabolite clustering results. **(C-D)** Pathway analysis comparison between lysine and vehicle (Veh) and glutamine (Gln), respectively. **(E)** Proposed influence of lysine treatment in *Pkd1*KO kidneys from metabolomic data. Metabolites in green are upregulated and red are downregulated with lysine compared to vehicle or glutamine treatment. Red dashed box symbolizes metabolites downregulated in females compared to males (*n* = 10/group). Created in BioRender. Sedaka, R. (2024) BioRender.com/c12s137. Results of partial least squares-discriminant analysis (PLS-DA) reported.

Kidney tissue from all three supplementation groups was further assayed to ascertain cellular metabolic changes. After filtering out false positives, there were a total of 67 positively and 66 negatively charged features annotated against an in-house 600 metabolite standard library (IROA Technologies, Sea Girt, NJ). Partial least squares-discriminant analysis (PLS-DA) was performed to compare group variation, finding that the selected principal components from each treatment group were distinct (Figure 5A). Clustering results show that annotated ions from lysine-supplemented mice distinctively cluster together compared to glutamine or vehicle groups (Figure 5B). The top upregulated metabolites in lysine-treated mice include proline, arginine, aminoadipate, glutarylcarnitine, citramalate, pyridoxamine and lysine, while the top downregulated metabolites include TCA cycle components succinate, fumarate, maleate, malate, and glutamate. L-carnitine, which converts to glutarylcarninitine during the conversion of glutaryl-CoA to glutaric acid, was also reduced. Pathway analysis comparing lysine to vehicle (Figure 5C) and lysine to glutamine (Figure 5D) revealed enrichment of lysine degradation and arginine biosynthesis, both of which increase aminoadipate and, subsequently, glutaryl-CoA. Because TCA cycle metabolites were low, it is likely that the conversion of glutaryl-CoA to acetyl-CoA is reduced and instead shifts toward glutaric acid production (Figure 5E).

**Figure 6.**
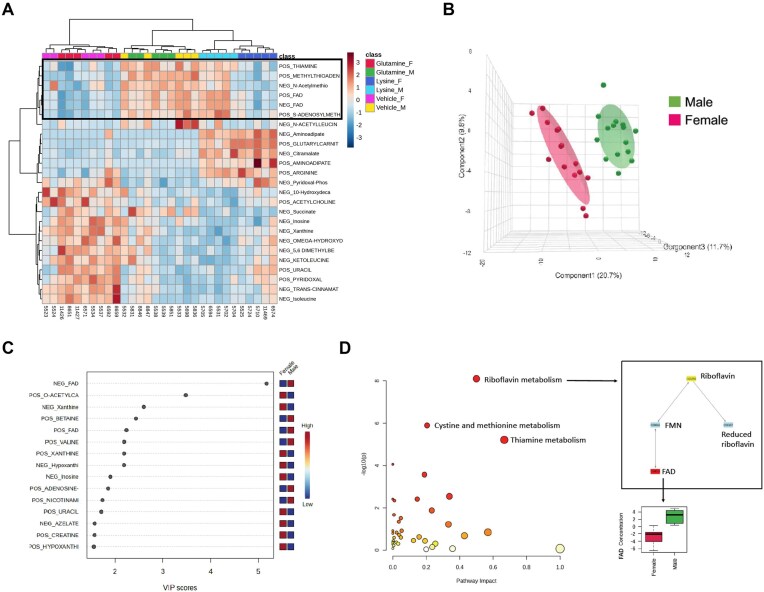
Metabolomic data reveals sex differences in *Pkd1*KO mice regardless of treatment. **(A)** Metabolite clustering results by OG and sex. **(B)** 3D scores plot between male and female *Pkd1*KO mice with variances shown in green versus pink colored ellipses, respectively. **(C)** Variable importance in projection (VIP) scores showing important features identified by PLS-DA. Colored boxes indicate the relative concentration of corresponding metabolites. **(D)** Pathway analysis comparison between male and female *Pkd1*KO mice, emphasizing the suppression of flavin adenine dinucleotide in females regardless of OG. (*n* = 15/sex). Results of PLS-DA reported.

Lastly, we clustered the metabolomic data by sex, which revealed an overarching difference between male and female mice regardless of supplementation (Figure 6A). The principal component analysis yielded distinct group variation by sex (Figure 6B), with the top differential metabolite being flavin adenine dinucleotide (FAD) (Figure 6C). Pathway analysis comparing male to female *Pkd1*KO mice showed that the top three pathways driving sex differences are riboflavin metabolism, which is associated with reduced FAD in female versus male mice, followed by cystine/methionine and thiamine metabolism (Figure 6D). Considering FAD is a cofactor in the mitochondrial electron transport chain,^37^ this reduction in FAD may correlate to the overall reduction in female *Pkd1*KO mouse kidney OCR as shown in Supplementary Fig. S2.

## Discussion

Dietary modifications are the most easily applied treatment plans to prevent rapid disease progression in PKD. The current study establishes that a plant-based WG diet mitigates kidney function decline, inflammatory agitation, and cyst-bearing effects of an animal-based casein diet in mice lacking *Pkd1*. Furthermore, supplementation with lysine induced kidney cyst growth and metabolic stress, which was not observed with glutamine supplementation.

Clinical data have shown that consuming more plant over animal protein is associated with better kidney function in patients with chronic kidney disease.^38,39^ Soy protein diet has been considerably studied in PKD, but revealed mixed results in slowing kidney cyst growth^21,23,25^ and initiated liver cyst growth in some models due to the high concentration of phytoestrogens.^24,26,40^ Wheat protein, which does not contain phytoestrogen, has been shown to suppress blood pressure spikes, kidney inflammation and kidney injury compared to casein protein in salt-sensitive Dahl rats on salt load.^27^ Since increases in inflammation promote cyst growth,^41,42,6^ we hypothesized that wheat-based protein may mitigate PKD progression. Indeed, our current study shows that high WG-fed *Pkd1*KO mice had (1) improved kidney function, as noted by lower plasma creatinine and *Kim1* expression, (2) less inflammation, as per fewer kidney resident macrophages and pro-inflammatory cytokines, and (3) smaller kidneys compared to HC-fed counterparts.

In establishing key differences between the diets, we first compared dietary composition. Despite a lower caloric intake, which is known to reduce kidney cyst burden in PKD,^5,43,44^ mice fed a high versus low protein diet had advanced cyst growth. Therefore, sugar, which was highest in the LC diet, does not stimulate cystogenesis in *Pkd1*KO mice. Next, we focused on differences in amino acid composition between the two high protein diets. We found that lysine, the most abundant amino acid in HC over WG diet, stimulated kidney cyst growth compared to glutamine, the most abundant amino acid in WG over HC diet, in *Pkd1*KO mice. Thus, we further investigated the role of lysine in worsening PKD.

Lysine is an essential amino acid that enters circulation via intestinal absorption^45^ and the kidneys via glomerular filtration. Once absorbed into renal epithelial cells, lysine is metabolized via the mitochondrial saccharopine pathway to ultimately produce glutaryl-CoA.^46^ We found that aminoadipate, an intermediary in this pathway, was upregulated in the kidneys of lysine-supplemented *Pkd1*KO mice (Figure 5D-E). Glutaryl-CoA is ordinarily converted to acetyl-CoA to fuel the TCA cycle, however, TCA cycle metabolites succinate, fumarate and malate were all downregulated with lysine supplement. There was likewise an increase in glutarylcarnitine, a by-product of glutaryl-CoA conversion to glutaric acid. Taking into account the observed acidemia and increased urinary ammonia excretion in lysine-supplemented mice, this indicates that lysine pushes glutaryl-CoA toward acid and away from energy production, ultimately causing metabolic stress and worsening kidney cysts. A dietary lysine load in a model of hypertension also resulted in increased lysine conjugate formation and reduced TCA cycle metabolites.^47^ Glutamine is utilized as an alternative fuel source for the impaired PKD TCA cycle to maintain cell homeostasis.^13-16^ Despite this essentiality, long-term glutamine supplementation did not result in increased cyst growth or overt metabolic changes compared to vehicle supplementation. Interestingly, glutamine was recently found to protect against acute kidney injury by blunting reactive oxygen species production and apoptosis, events known to slow PKD progression.^48^

Notably, both lysine and glutamine supplementation increased GFR compared to vehicle, but only lysine supplementation increased kidney cystic index. This suggests that higher GFR, per se, is not an independent stimulator for accelerated cystogenesis. This was surprising since both the unilateral nephrectomy^7^ and high protein diet^12^ hypertrophy models displayed increases in GFR and accelerated cystogenesis in PKD mice. A limitation of the present study is that other amino acids known to stimulate cyst growth and/or GFR in PKD, including arginine, branched-chain amino acids, methionine and tryptophan,^17-20^ were not compared to lysine and glutamine. However, it was recently reported that lysine is one of the top amino acids found in both cystic fluid and urine in PCK rats.^49^ Lysine is generally low in wheats and fruits and high in meat, poultry and dairy.^50^ A study in healthy humans fed a meat rich compared to a vegan diet display a positive correlation with serum lysine levels and a negative correlation with serum glutamine levels.^51^ Although these studies insinuate a negative impact of lysine on cyst growth, future studies are necessary to tease out whether lysine reaches the cystic epithelium via dietary lysine-tracing and whether long-term low lysine diet would slow PKD.

Finally, we acknowledge the sex differences in our mice. Male *Pkd1*KO mice had significant reductions in kidney cyst burden and overall immune response on a high WG compared to casein protein diet, but this effect was nonexistent in females. Although we cannot definitively pinpoint the cause of these sex differences, our study shows that female *Pkd1*KO mice had lower kidney mitochondrial function (Supplementary Fig. S2) and reduced flavin adenine dinucleotide (FAD) (Figure 6) compared to males. FAD is a strong oxidizing agent involved in the TCA cycle, electron chain transport, fatty acid oxidization, and amino acid catabolism.^52^ Patients with glutaric aciduria type II, an inherited FAD disorder, can exhibit fatty acid oxidation dysfunction, acidosis and polycystic kidney disease.^53^ Therefore, FAD and mitochondrial dysregulation in female mice may be associated with increased cystogenesis, which could explain why females were resistant to the benefit of WG diet. This postulation is limited by the fact that the two results being compared are from separate study cohorts, and therefore no statistical associations can be drawn.

In summary, an animal compared to plant-based protein diet accelerates kidney growth in PKD. Increased intake of lysine, which is abundant in animal-based protein, leads to metabolic stress and worsened PKD progression. While the advantages of WG in patients remains unknown, wheat protein is safe and changing protein source instead of protein load could be a meaningful long-term dietary intervention in PKD.

## Supplementary Material

zqaf026_Supplemental_File

## Data Availability

Metabolomic data are available at the NIH Common Fund's National Metabolomics Data Repository website, the Metabolomics Workbench (https://www.metabolomicsworkbench.org),^54^ where it has been assigned Study ID ST003518. The data can be accessed directly via its Project DOI: http://dx.doi.org/10.21228/M81J94. This work is supported by NIH grant U2C-DK119886 and OT2-OD030544 grants. All other data generated and analyzed in the current study are available from the corresponding author upon reasonable request.
