# Supplementary material for: Wheat-Based Protein Slows Disease Progression in Pkd1 Knockout Mice
Source: Function (Oxf). 2025 Jun 10;6(4):zqaf026. doi: 10.1093/function/zqaf026 (PMC12342364; doi:10.1093/function/zqaf026)
Supplement: zqaf026_Supplemental_File [file zqaf026_supplemental_file.docx]

**Supplemental Materials**

**Wheat-based protein slows cyst growth in *Pkd1* knockout mice**

Randee Sedaka^1^, Jifeng Huang^1^, Shinobu Yamaguchi^1^, Emily Hallit^1^, Aida Moran-Reyna^1^, Jung-Shan Hsu^1^, Caleb Lovelady^1^, Ayaka Fujihashi^1^, Mohammad Sako^2^, Malgorzata Kasztan^2^, Gloria  Benavides ^3^, Landon Wilson^4^, Victor Darley-Usmar^3^, Stephen Barnes^4^, Takamitsu Saigusa^1*^

^1^Section of Cardio-Renal Physiology and Medicine, Division of Nephrology, Department of Medicine, University of Alabama at Birmingham, Birmingham, AL, United States

^2^Division of Pediatric Hematology/Oncology, Department of Pediatrics, University of Alabama at Birmingham, Birmingham, AL, United States

^3^Department of Pathology, University of Alabama at Birmingham, Birmingham, AL, United States

^4^Targeted Metabolomics and Proteomics Laboratory, University of Alabama at Birmingham, Birmingham, AL, United States

Corresponding Author: Takamitsu Saigusa, MD

**Table of Contents**

**Supplementary Methods** pg 3-4

**Supplementary Table 1.** Nutritional composition of diets pg 5

**Supplementary Table 2.** Amino acid composition of diets pg 5

**Supplementary Table 3.** Antibodies used for Flow Cytometry pg 6

**Supplementary Table 4.** Primers for quantitative RT-PCR pg 6

**Supplementary Figure 1.** Females are not responsive to WG diet pg 7

**Supplementary Figure 2.** Sex differences in OCR pg 8

**SUPPLEMENTARY** **METHODS**

**Liquid Chromatography Tandem Mass Spectrometry (LC-MS/MS)**

***Sample Preparation:*** Kidney cortex tissue (~100 mg) was bead homogenized in 500 μL of 4^o^C 1X PBS using a NextAdvance system (Troy, New York). An aliquot of homogenate (100 μL) was added to ice-cold PBS (100 μL) and subjected to a liquid-liquid extraction procedure (Bligh-Dyer Protocol). Methanol:chloroform (2:1, v/v, 750 μL) was added to each sample, followed by chloroform (250 μL) and then ddH_2_0 (250 μL). After vigorous mixing, samples were centrifuged at 1750 x g for 10 minutes at 4^o^C to separate the two phases. The upper aqueous layer containing water-soluble metabolites was transferred to a new glass test tube and dried under N_2_. Samples were then re-suspended in ice-cold 80% aqueous methanol (500 μL) to precipitate proteins and centrifuged at 1750 x g for 10 minutes at 4^o^C. Supernatants were transferred to a new tube and dried under N_2_. Each sample was re-suspended in ddH_2_0 (200 μL) containing 0.1% formic acid for mass spectrometry analyses.

***LC-MS/MS Analysis (Untargeted Metabolomics):*** For each specimen analyzed, an aliquot (10 μL) was loaded onto a Phenomenex 2.1 i.d. x 100 mm, 1.6 μm Luna Omega, 80 Å reverse-phase column (Torrance, CA) equilibrated with 0.1% formic acid. Each sample was subjected to a linear gradient of 2-50% mobile phase B for 5 min, 50-98% B until 6 min with a 1-minute hold, and then re-equilibrated at initial conditions for 3 minutes using an Exion UHPLC (Sciex, Concord, Ontario, Canada) at a flow rate of 500 μL/min. The mobile phases were A) ddH_2_O with 0.1% formic acid and B) acetonitrile with 0.1% formic acid. A Sciex 5600 Triple-Tof mass spectrometer was used to analyze the metabolite profile. The IonSpray voltages for positive and negative modes were +5500/-4500 V, respectively, and the declustering potential was +/- 80 V. Ionspray GS1/GS2 and curtain gases were set at 40 psi and 25 psi, respectively. The interface heater temperature was 400^o^C. Eluted compounds were subjected to a time-of-flight survey scan from *m/z* 50-1000 only.

A pooled sample containing equal volumes from each biological specimen was created to analyze for metabolite annotations and to determine instrument stability. The mass spectrometer conditions were identical to the analyzed plasma/sera specimens except for the addition of data-dependent scans to select the top eight most intense precursor ions for MS/MS analysis. Product ion time-of-flight MS/MS scans were over *m/z* 50-1000 and were collected at 50 msec intervals using a collision energy spread of 15 eV with a set collision point of 35 eV. Spectra were centroided and de-isotoped by Analyst software, version 1.81 TF (Sciex, Toronto, Canada).

***Data Analysis and Metabolite Annotation:*** LC-MS data were processed using MS-Dial 4.90 (RIKEN Center, Yokohama City, Kanagawa, Japan) to identify peaks occurring across all samples and to determine peak areas and retention times. Metabolites were annotated using the IROA 600 metabolite standards library (IROA Technologies, Sea Girt, NJ) that were analyzed under the same LC-MS conditions as the samples. Each annotated metabolite was verified by evaluating fragmentation spectra using PeakView 2.2 software (SCIEX). A processed .csv file was uploaded to MetaboAnalyst 6.0 (<https://www.metaboanalyst.ca/>) for statistical evaluations.

**Supplementary Table 1.** Nutritional composition of diets

**Supplementary Table 2.** Amino acid composition of diets

**Supplementary Table 3.** Antibodies used for Flow Cytometry

**Supplementary Table 4.** Primers for quantitative RT-PCR

**
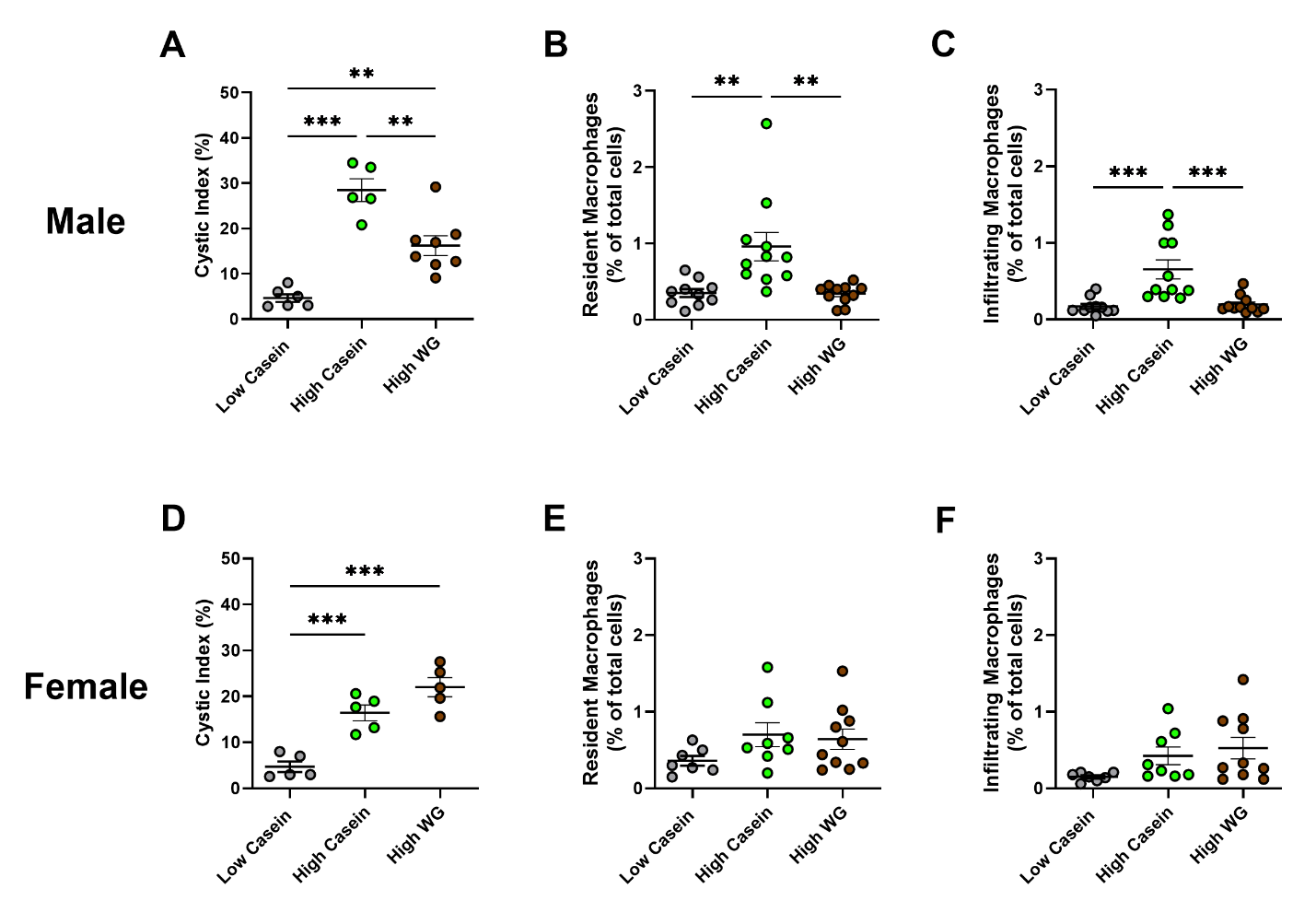
**

**Supplementary Figure 1. Females are not responsive to WG diet. (A)** Male cystic index, **(B)** number of resident macrophages, and **(C)** number of infiltrating macrophages. High casein diet increases cystic index and numbers of both macrophages compared to low casein diet, but high WG is protective against these increases. **(D)** Female cystic index, **(E)** number of resident macrophages, and **(F)** number of infiltrating macrophages. While female *Pkd1*KO mice have increased cystic index in response to a high protein load, source does not impact this index. Females were protected from changes in macrophage number. (n= 5-11/group). Results of a One-way ANOVA with Tukey’s multiple comparisons test reported for all. *P* **< 0.01, ***< 0.001.

**
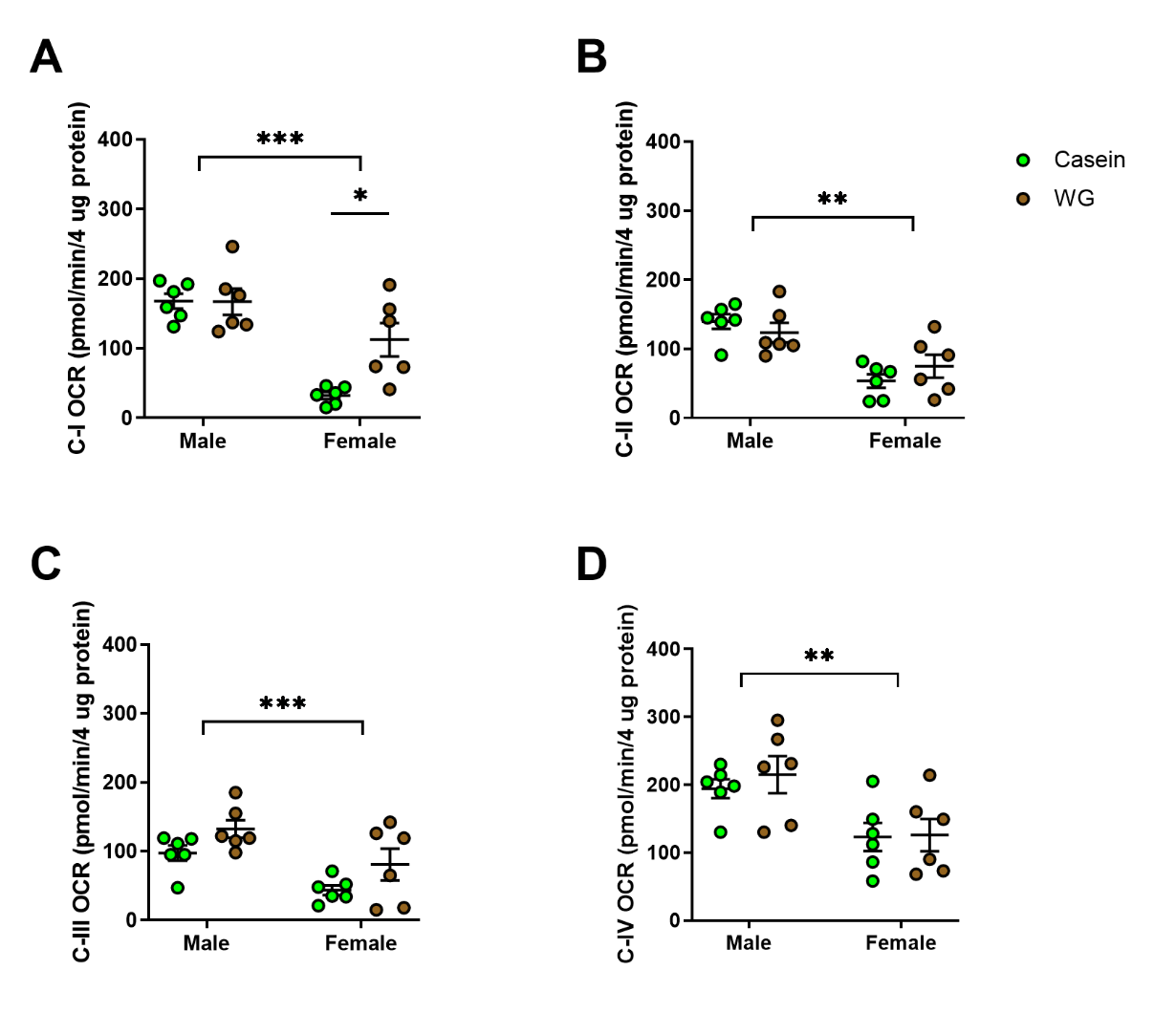
**

**Supplementary Figure 2. Sex differences in OCR. (A)** Mitochondrial complex I, **(B)** complex II, **(C)** complex III, and **(D)** complex IV OCR between male and female *Pkd1*KO mice fed high casein or WG diet for 6 weeks. Overall, females had a lower OCR than males irrespective of diet. WG increased the OCR of complex I over casein diet in female mice only. (n= 6/group). Results of a two-way ANOVA with Tukey’s multiple comparisons test reported for all. *P* *< 0.05, **< 0.01, ***< 0.001.
